# Targeting the Oncogenic TBX2 Transcription Factor With Chromomycins

**DOI:** 10.3389/fchem.2020.00110

**Published:** 2020-03-03

**Authors:** Bianca Del B. Sahm, Jade Peres, Paula Rezende-Teixeira, Evelyne A. Santos, Paola C. Branco, Anelize Bauermeister, Serah Kimani, Eduarda A. Moreira, Renata Bisi-Alves, Claire Bellis, Mihlali Mlaza, Paula C. Jimenez, Norberto P. Lopes, Glaucia M. Machado-Santelli, Sharon Prince, Leticia V. Costa-Lotufo

**Affiliations:** ^1^Department of Pharmacology, Institute of Biomedical Sciences, University of São Paulo, São Paulo, Brazil; ^2^Division of Cell Biology, Department of Human Biology, University of Cape Town, Cape Town, South Africa; ^3^Department of Cell and Developmental Biology, Institute of Biomedical Sciences, University of São Paulo, São Paulo, Brazil; ^4^Department of Physics and Chemistry, School of Pharmaceutical Sciences, University of São Paulo, Ribeirao Preto, Brazil; ^5^Department of Sea Sciences, Federal University of São Paulo, Santos, Brazil

**Keywords:** DNA-binding agents, T-box factors, reverse affinity, microscale thermophoresis, melanoma, chromomycins

## Abstract

The TBX2 transcription factor plays critical roles during embryonic development and it is overexpressed in several cancers, where it contributes to key oncogenic processes including the promotion of proliferation and bypass of senescence. Importantly, based on compelling biological evidences, TBX2 has been considered as a potential target for new anticancer therapies. There has therefore been a substantial interest to identify molecules with TBX2-modulatory activity, but no such substance has been found to date. Here, we adopt a targeted approach based on a reverse-affinity procedure to identify the ability of chromomycins A_5_ (CA_5_) and A_6_ (CA_6_) to interact with TBX2. Briefly, a TBX2-DNA-binding domain recombinant protein was N-terminally linked to a resin, which in turn, was incubated with either CA_5_ or CA_6_. After elution, bound material was analyzed by UPLC-MS and CA_5_ was recovered from TBX2-loaded resins. To confirm and quantify the affinity (K_D_) between the compounds and TBX2, microscale thermophoresis analysis was performed. CA_5_ and CA_6_ modified the thermophoretic behavior of TBX2, with a K_D_ in micromolar range. To begin to understand whether these compounds exerted their anti-cancer activity through binding TBX2, we next analyzed their cytotoxicity in TBX2 expressing breast carcinoma, melanoma and rhabdomyosarcoma cells. The results show that CA_5_ was consistently more potent than CA_6_ in all tested cell lines with IC_50_ values in the nM range. Of the cancer cell types tested, the melanoma cells were most sensitive. The knockdown of TBX2 in 501mel melanoma cells increased their sensitivity to CA_5_ by up to 5 times. Furthermore, inducible expression of TBX2 in 501mel cells genetically engineered to express TBX2 in the presence of doxycycline, were less sensitive to CA_5_ than the control cells. Together, the data presented in this study suggest that, in addition to its already recognized DNA-binding properties, CA_5_ may be binding the transcription factor TBX2, and it can contribute to its cytotoxic activity.

## Introduction

Traditionally, drug discovery and development (D&D) programs have made use of phenotypic approaches, which are characterized by observable changes in a disease model (animal or cellular). Advances in molecular biology and biochemistry, as well as the sequencing of the human genome, has enabled a more reductionist and rational approach to D&D. It has made possible the principle of targeting molecular drivers of diseases as well as increasing and improving approaches to targeting these molecules (Strausberg and Schreiber, 2003; Overington et al., 2006; Swinney and Anthony, 2011). In this context, designing targeted-based drug screening protocols with integrated strategies, is an attractive point to start drug discovery programs.

Affinity-based methods have gained attraction as innovative approaches to targeted oriented drug discovery. These involve the use of a molecular probe that is specifically designed to identify one or more target proteins from many others present in a complex cell lysate mixture. The identification and quantification of the isolated targets are ascertained by liquid-chromatography coupled to tandem mass spectrometry (LC-MS) (Rylova et al., 2015). Using the reverse of this approach, Lau et al. (2015) screened natural crude extracts for compounds with affinity for a specific biological protein that had previously been characterized and, hence, proposed as an important therapeutic target. Briefly, in this approach, the target protein was attached to an affinity resin, exposed to complex natural extracts and compounds that were bound to the protein were then detected by LC-MS.

The establishment of new targets in cancer therapy is a key step in the development of new effective therapies. In this realm, the transcription factor TBX2 overexpression has been directly linked to several cancers, including rhabdomyosarcoma (Zhu et al., 2016), breast (Jacobs et al., 2000; Redmond et al., 2010), melanoma (Vance et al., 2005; Peres et al., 2010), nasopharyngeal (Lv et al., 2017), and prostate cancers (Du et al., 2017). Indeed, TBX2 functions as a potent growth-promoting factor, in part due to its ability to bypass senescence and to repress key negative cell cycle regulators such as p14ARF, p21, and NDRG (Jacobs et al., 2000; Prince et al., 2004; Redmond et al., 2010). Furthermore, there is strong *in vitro* and *in vivo* biological evidence that TBX2 may be a novel target for anti-cancer drugs that can be administered on their own or in combination with other chemotherapies. Indeed, knocking down TBX2 in melanomas or in several metastatic breast cancer cell lines resulted, respectively, in induction of senescence or in a profound inhibition of proliferation, regardless of their receptor status (Peres et al., 2010; Wansleben et al., 2014). TBX2 also confers resistance to the widely used chemotherapeutic drug cisplatin by promoting p53 activity via Chk2, further leading to an S-phase arrest and DNA repair. Importantly, depleting TBX2 sensitizes cisplatin-resistant breast cancer and metastatic melanoma cells to this drug. These results suggest that TBX2 stimulates proliferation and inappropriate survival of cells with damaged DNA (Wansleben et al., 2013). Any drug that therefore impacts TBX2 expression or activity is likely to have a major impact on cancer progression and recurrence.

Natural products have provided an important source of bioactive molecules for clinical use. They are extensively used in the pharmaceutical industry as either drugs or in influencing the synthesis and semisynthesis of therapeutic molecules. Of particular importance, ~60% of anti-cancer agents currently in the clinic are derived from natural products (Newman and Cragg, 2016). DNA-binding agents are the most common class of anticancer drugs. They function by interacting with DNA of dividing cells, resulting in DNA-damage and, thus, blocking transcription and replication that, ultimately, halts the cell cycle and/or activates cell death pathways. They can also inhibit enzymes that are important for the maintenance of DNA integrity such as topoisomerases. Chromomycins are tricyclic glycosylated polyketides belonging to the aureolic acid family, with promising anticancer activity and antiproliferative properties (Guimarães et al., 2014; Pettit et al., 2015; Pinto et al., 2019). These molecules bind to the DNA minor groove, causing DNA damage of treated cells, enhancing the expression of apoptosis related genes (Boer et al., 2009; Zihlif et al., 2010).

In this context, the present study employed a reverse affinity approach using the DNA-binding domain of the anticancer target TBX2 as bait to access the potential affinity of the marine chromomycins CA_5_ and CA_6_. Microscale thermophoresis was applied to quantify the binding affinities of the natural compounds, CA_5_ and CA_6_, to TBX2. Cytotoxicity of these compounds were determined in TBX2-driven breast carcinoma, melanoma, and rhabdomyosarcoma cell lines.

## Materials and Methods

### Reagents

**Figure 1:**
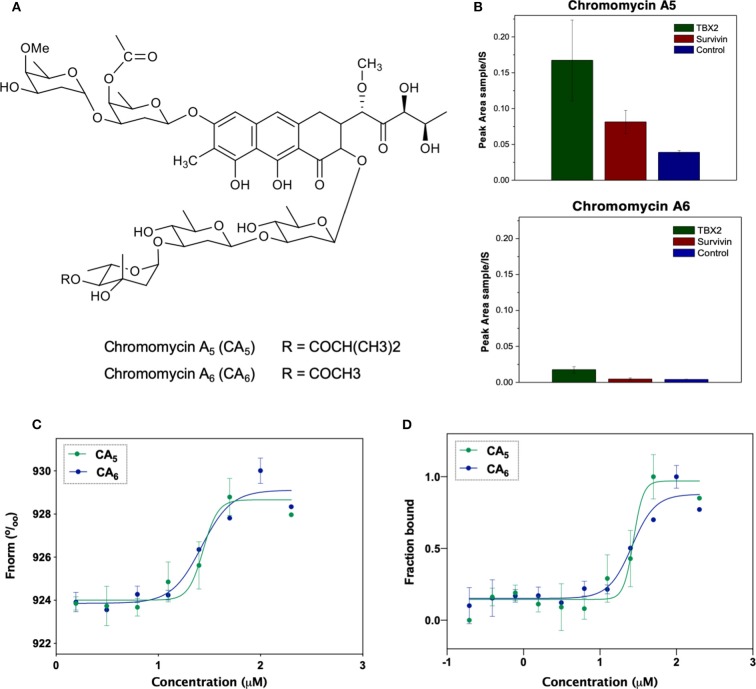
**(A)** Chemical structures of the compounds used in the present study: chromomycin A_5_ and chromomycin A_6_. **(B)** Relative quantification of compounds chromomycin A_5_ and chromomycin A_6_ recovered from the bioaffinity chromatography technique using resins functionalized with TBX2 (green) or Survivin (red), or non-functionalized (control, blue) resin. The value of peak areas (triplicate) was used to construct the graphic. **(C)** Binding affinities of test compounds to TBX2 assessed through microscale thermophoresis. Normalized fluorescence of labeled TBX2 in the presence of serial concentrations of each of the test molecules. Data correspond to the mean values from two independent experiments. **(D)** Bound fraction of fluorescently labeled TBX2 with serial concentrations of each of the two chromomycins CA_5_ and CA_6_.

The isolation and characterization of chromomycins A_5_ (CA_5_) and A_6_ (CA_6_) was previously described by Pinto et al. (2019). The substances were resuspended in dimethyl sulfoxide (DMSO, Sigma Aldrich, USA). Figure 1A shows the chemical structure of CA_5_ and CA_6_.

### Cell Culture

The human melanoma cells lines 501mel, MM200, and WM293a (kindly donated by Professor Dorothy Bennet, St George's University of London) were cultured in Roswell Park Memorial Institute Medium (RPMI)-1640 (Sigma Aldrich, USA). The human breast adenocarcinoma cell lines MCF-7 (ATCC® HTB-22) and T47D (ATCC® HTB-133) were cultured in Dulbecco's Modified Eagle medium:nutrient mixture F-12 (Ham) (DMEM/F-12) (Gibco by Thermo Fisher Scientific, USA). The human embryonal rhabdomyosarcoma cell line RD (ATCC® CCL-136) and the human alveolar rhabdomyosarcoma cell line RH30 (kindly provided by Professor Judith Davie, Southern Illinois University) were cultured in DMEM (Sigma Aldrich, USA). All culture medium was supplemented with 10% heat-inactivated fetal bovine serum (FBS), 100 U/mL penicillin and 100 μg/mL streptomycin. Cells were maintained at 37°C in a 5% CO_2_ humidified incubator and medium was replaced every 2–3 days.

### TBX2 Protein Expression and Purification

The coding region of the human TBX2 DNA binding domain (residues 94–287) was amplified by PCR and cloned into a pET28b vector between Nde1 and Xho1 restriction sites to generate an N-terminal His6-tagged sequence containing a thrombin cleavage site. The construct was verified by DNA sequencing. The pET28b-TBX294-287 plasmid was transformed into *Escherichia coli* strain BL21(DE3) and expression carried out at 37°C for 4 h, resulting in a 215-amino acid protein with a molecular weight of 24 kDa. The protein was purified by immobilized nickel affinity chromatography (GE Healthcare Life Sciences, USA); using HisTrap HP nickel affinity column (GE Healthcare Life Sciences, USA), with binding in a buffer containing 20 mM sodium phosphate buffer (pH 7.5), 500 mM NaCl and 50 mM imidazole and elution in the same buffer with a 50–300 mM imidazole gradient. The protein was further purified by size exclusion chromatography on a Superdex 200 column (GE Healthcare Life Sciences, USA) in a Reverse Affinity (RA) buffer containing 20 mM HEPES pH 7.4, 150 mM KCl, 1 mM MgCl_2_, 2 mM β-mercaptoethanol (BME). Purity of the protein was verified by SDS-PAGE and protein concentration was determined using NANODROP 2000 system (Thermo Fisher Scientific, USA).

Survivin protein was included in the reverse-affinity chromatography assay as negative protein control. The survivin plasmid was obtained from Professor Eli Chapman (Department of Pharmacology and Toxicology, University of Arizona, USA), containing the full-length protein with an extra kanamycin-resistant histidine tail (His-tag). The plasmid was transformed into the *E. coli* strain BL21(DE3) and expression carried out at 16°C overnight. The survivin protein was purified as described for the TBX2 DNA binding domain. After removing the non-specific binders with wash buffer (lysis buffer + 2 mM BME) and complete astringent washing (50 mM Hepes pH 7.4, 1 M KCl, 5 mM MgCl_2_, 2.5% glycerol in ddH_2_O + 2 mM BME), elution buffer (50 mM Hepes pH 7.4, 150 mM KCl, 5 mM MgCl_2_, 5% glycerol, 250 mM Imidazole in ddH_2_O) was added. The protein was further purified overnight by dialysis using Spectra/Por dialysis membrane of 3.5 kD (Spectrum Labs Inc., USA) and dialysis buffer (20 mM Hepes pH 7.4, 150 mM KCl, 1 mM MgCl_2_ in ddH_2_O), then concentrated in Amicon Ultra-15 10 K (Merck-Millipore, USA) centrifuge filters at 4,000 rpm at 4°C. Purity of the protein was verified as described for TBX2 DNA binding domain.

### Reverse Affinity Procedure

The samples obtained from the bioaffinity chromatography (triplicate) were solubilized with 0.5 mL of methanol containing 0.2 mg/mL of chlorogenic acid (3-caffeoylquinic acid) used as internal standard (IS). Samples were filtered in a small column (made in house on the tip of a Pasteur pipette) containing Sephadex LH-20 in order to remove the salt scraps from buffer. Filtered samples were analyzed by UPLC-MS/MS method on an Acquity TQD (WatersR Corporation, USA) instrument equipped with an ultra-performance liquid chromatography (UPLC) system coupled to a mass spectrometer fitted with an electrospray ionization source and a triple-quadrupole MS detector. Twenty microliter were injected into a Kinetex—Core-Shell Technology C18 column (100 A, 50 × 2.1 mm, 1.7 μm; Phenomenex, USA) kept at 40°C. The mobile phase consisted of deionized water (phase A) and methanol (phase B), both containing 0.2% of formic acid. The chromatographic condition applied was as follows: 5% phase B at 0 min, 100% phase B at 6 min, 100% phase B at 8 min, and 5% phase B at 10 min. The flow rate used was 0.2 mL/min. The ionization source conditions were as follows: 3.0 kV capillary voltage; 20 V cone voltage; 150°C source temperature; 300°C desolvation temperature; 500 L/h desolvation gas flow; 50 L/h cone gas flow. Data was acquired in positive ionization mode employing selected ion recording (SIR) mode. The peak area obtained for the compounds in each sample was normalized by the peak area of the IS in the same run, which allows the quantitative comparison between different sample (obtained from different proteins) for the same compound.

### Microscale Thermophoresis

Binding affinities between target proteins and ligands were measured using microscale thermophoresis (MST) according to the NanoTemper technologies protocol in a Monolith NT.115 (Nanotemper Technologies, Germany) (Duhr and Braun, 2006). Proteins were fluorescently labeled using the Monolith Protein Labeling Kit RED-NHS 2nd Generation (Amine Reactive) (Nanotemper Technologies, Germany) according to the manufacturer's' instructions. The experiments were performed in three independent replicates using Monolith NT.115 Premium glass capillaries (Nanotemper Technologies, Germany) and driven in RA buffer with final concentration of 2% DMSO and 0.05% Tween 20. PCR microtubes were prepared with ligands and the target protein solutions. Ligand concentrations ranged from 10 nM to 400 μM while protein concentrations remained constant. After 5 min at room temperature, the samples were loaded into Premium glass capillaries and the experiments were performed using 20 and 40% MST power and between 20 and 80% LED power at 24°C. MST traces were recorded using the standard parameters: 5 s MST power off, 30 s MST power on and 5 s MST power off. The binding affinities of the compounds to the protein were determined according to with dissociation values (Kd) and the generated data were processed using MO. Control software (Nanotemper Technologies, Germany).

### Western Blot Analyses

Rhabdomyosarcoma cells were lysed in whole cell lysis buffer (0.125 M Tris-HCl pH 6.8, 4% SDS, 0.2% glycerol, 0.1% BME, and a pinch of bromophenol blue) and boiled for 10 min and the remaining cell lines were lysed in RIPA buffer [50 mM Tris–HCl (pH 7.5), 150 mM NaCl, 0.1% NP-40, 0.5% sodium deoxycholate, 1 mM EDTA, and 2 mM EGTA] containing 1 mM sodium orthovanadate, 1 mM phenylmethylsulfonyl fluoride, 10 mg/ml leupeptin and 10 mg/ml aprotinin. Equal amounts of proteins were resolved by SDS-PAGE (12% gels) and transferred to Hybond ECL (Amersham Biosciences, UK) or nitrocellulose (Bio Rad Laboratories, USA) membranes. The membranes were incubated with goat polyclonal antibody to TBX2 (sc-17880) from Santa Cruz Biotechnology (USA) for rhabdomyosarcoma cells, or with rabbit polyclonal antibody to TBX2 (16930-1-AP) from Proteintech (USA) for melanoma and breast carcinoma cells. Mouse monoclonal antibody antiFlag M2 (F1804) from Sigma-Aldrich (USA) was used in the genetically engineered 501mel cells experiments. To control for loading, the rabbit polyclonal antibody to p38 MAPK (#9212) from Sigma-Aldrich (USA), or α-tubulin rabbit polyclonal antibody (#2144) or β-actin rabbit monoclonal antibody to (#4970) from Cell Signaling Technology (USA) were used. After incubation with primary antibodies, membranes were washed then incubated with HRP-conjugated secondary antibodies (#7074 Cell Signaling Technology, USA). Antibody reactive proteins were visualized by enhanced chemiluminescence using SuperSignal West Pico Chemiluminescent Substrate Kit (Thermo Fisher Scientific, USA) or WesternBright ECL HRP Substrate Kit (Advansta Company Inc., USA). Densitometry readings were obtained using UN-SCAN-IT gel 6.1 software (Silk Scientific, USA) and protein expression levels were represented as ratio signals for TBX2/respective loading control. All blots are representative of at least two independent repeats.

### Cell Viability Assays

Cells were seeded in 96-well-plates and, in the following day, they were treated with a range of CA_5_ and CA_6_ concentrations or vehicle (1.0 μL DMSO) for 48 or 72 h. Cell viability was measured using the 3-(4,5-dimethylthiazol-2-yl)-2,5-diphenyl-trazolium bromide (MTT) assay (Invitrogen by Thermo Fisher Scientific, USA) (Mosmann, 1983). Mean cell viability was calculated as a percentage of the mean vehicle control. Three independent experiments were performed from which the half maximal inhibitory concentration (IC_50_) and their respective 95% CI (confidence interval) were obtained by non-linear regression using GraphPad Prism version 8.0 (GraphPad Software, USA).

### TBX2 Knockdown (shTBX2) 501mel Cells

To generate stably transfected cell lines in which TBX2 mRNA levels are knocked down (shTBX2), oligonucleotides targeting 5′-ACAGCTGAAGATCGACAACAA-3′ (TBX2 siRNA) of the human coding sequences was cloned into the pSuper.neo/GFP (Oligoengine) shRNA expression vector. The non-specific siRNA oligonucleotide (shControl) was directed against a 5′-ATTTCTCCGAACGTGTCACGT-3′ target sequence. 501mel cells were transfected with the pSuper.neo/GFP expression vector containing sequences targeted to TBX2 or the non-specific control using Transfectin® (BioRad, Hercules, CA). Four hundred microgram per milliliter of G418 disulfate salt (Sigma Aldrich, A1720) were used to maintain and select transfected cells (Webster and Dickson, 1983). Prior to experiments, cells were probed for TBX2 by Western blot to confirm protein knockdown relative to parental- and shControl-cell levels. Cytotoxicity of CA_5_ was compared in these different 501mel cell models using the MTT assay following 72 h incubation. Therefore, three independent experiments were performed in duplicates from which the IC_50_ was obtained by non-linear regression using GraphPad Prism version 8.0 (GraphPad Software, USA).

### FLAG-Tagged TBX2 (iTBX2) 501mel Cells—Rescue Experiments

501mel cells were genetically engineered to allow for inducible expression of 3XFLAG-tagged TBX2 (iTBX2) using a tetracycline-on (Tet-On) system (kindly provided by Professor Colin Goding, Ludwig Institute of Cancer, Oxford University, UK). TBX2 was induced using 60 ng/mL (135 nM) of doxycycline (Das et al., 2016). TBX2 was induced using 60 ng/mL (135 nM) of doxycycline either 12 h before, or 12 h following, drug treatments with CA_5_. An iTBX2-empty cell line was used as an experimental control. Cells were exposed to serial concentrations of either drug, and the end point for both conditions was at 48 h after drug exposure. IC_50_ for cell models subjected to both experimental conditions and drug treatments were obtained using the MTT assay and calculated from three independent experiments performed in duplicates by non-linear regression using GraphPad Prism version 8.0 (GraphPad Software, USA).

## Results and Discussion

### Chromomycins Directly Interact With the TBX2 DNA Binding Domain

Our group has been focused on screening and developing natural products with anticancer potential. In order to optimize and test new strategies in this field, we are currently applying the reverse affinity procedure as a tool to screen and guide the isolation of target-specific anticancer substances within crude bacterial extracts. In this context, the oncogenic TBX2 transcription factor appears as an intriguing target due to its important pro-tumor roles and the fact that no known modulator was discovery so far. In fact, the DNA binding agent trabectedin (Yondelis®) was the first compound able to displace an oncogenic transcription factor from its target promoters with high specificity (D'Incalci et al., 2014). Moreover, a comparable cytotoxicity profile of trabectedin and chromomycin A_3_, as assessed in the COMPARE analysis using data from NCI 60 cell line panel, point to a similar mechanism of action (Marco and Gago, 2005). Therefore, pondering on these evidences, we first attempted to verify whether the chromomycins A_5_ and A_6_–previously isolated by our group from a marine bacteria *Streptromyces* sp. BRA384—showed affinity to the TBX2 DNA binding domain.

The analyses of samples recovered from the reverse affinity procedure (Figure 1B) shows that both compounds displayed a residual binding to the resin itself and to either of the protein-loaded resins, however the recovery of CA_5_ was increased by the presence of TBX2 linked to the resin. Although this technique has been described as a cost-effective procedure to identify binding compounds based on their affinity properties to a target protein (Lau et al., 2015), further analyses are necessary to validate the observed interactions. Actually, multiple unspecific interactions could be expected due to the ability of natural products to interact with proteins (Clardy and Walsh, 2004) and, moreover, binding to the target does not necessarily turn out in any biological modulation. Herein, we describe, for the first time, that this procedure can also be applied to screen modulators of transcription factors, such as the TBX2 DNA-binding domain as target.

In order to characterize the binding affinity between TBX2 and chromomycins, we used microscale thermophoresis (MST). This technique characterizes a ligand-binder interaction based on the movement of a given protein along a temperature gradient (thermophoresis), which, in turn, depends on its molecular size, charge and hydration shell. Once a ligand is bound to the protein, a distinct thermophoretic movement is expected due to a change in at least one of the above-mentioned parameters, and the information is compared between unbound and bound states (Wienken et al., 2010; Mueller et al., 2017). Figure 1C shows that CA_5_ and CA_6_ were able to change the thermophoretic movement of TBX2. The dissociation constants (K_D_) for interactions were calculated for each chromomycin by measuring changes in the fluorescently labeled TBX2 by thermophoresis upon compound binding then normalizing and plotting this as a function of the ligand concentration (Figure 1D). The results suggest that CA_5_ and CA_6_ exhibit similar binding affinities to the TBX2 DNA-binding domain, with K_D_ of 31.3 ± 23.3 and 24.4 ± 13.6 μM, respectively.

Most of the available reports on this class of molecules regard the biological properties of CA_3_ and, to a less extent, of CA_2_. These chromomycins are, in turn, stereoisomers of CA_6_ and CA_5_, respectively, as demonstrated by Pinto et al. (2019) and Pettit et al. (2015). CA_3_ binds to the minor groove of the DNA complex as a dimer in a process dependent on Mg^+2^, in which a portion of the chromophore along with the D, E and F sugar moieties interact with residues of the minor groove, while the A and B sugar moieties and another portion of the chromophore interact with the phosphate backbone (Gao and Patel, 1989; Boer et al., 2009). Moreover, studies on the interaction of CA_3_ and DNA revealed an apparent affinity constant in the micromolar range (Behr et al., 1969; Aich et al., 1992). To the best of our knowledge, there is no data on the interaction of CA_5_ and CA_6_ with DNA. Nonetheless, the structural similarities among this class of compounds suggest that DNA-binding properties should be expected. Further studies are necessary to verify whether these molecules are directly binding to DNA and, furthermore, whether the interaction of TBX2 with DNA could be altered in the presence of chromomycins.

### CA_5_ Displays Potent Cytotoxicity Against Different TBX2-Driven Cancer Cell Lines

**Figure 2:**
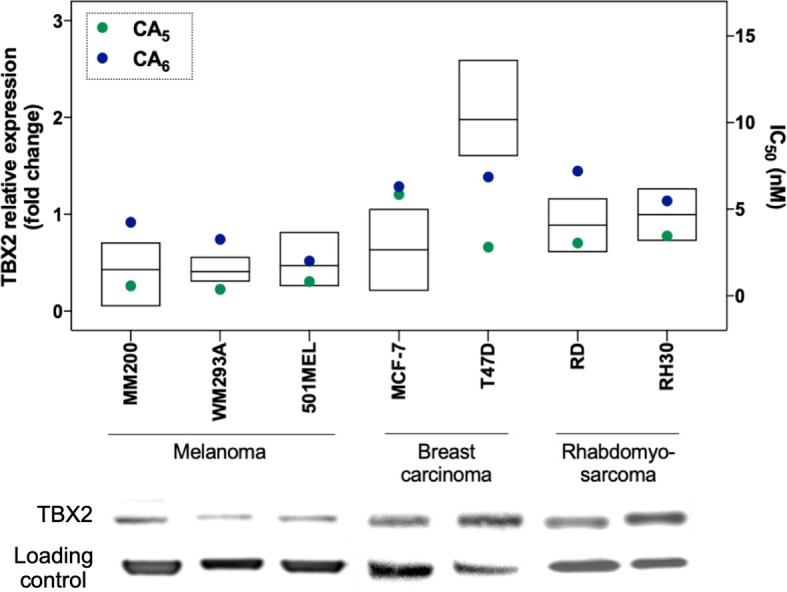
Relative protein expression of TBX2 and cytotoxicity (IC_50_) data in melanoma, breast cancer, and rhabdomyosarcoma cell lines. Protein expression levels are plotted in the left y axis as ratio signals for TBX2/respective loading control obtained from the densitometry readings using UN-SCAN-IT gel 6.1 software. Loading controls were obtained using α-tubulin for melanoma and breast carcinoma cells, and P-38 for sarcoma cells. IC_50_ values are represented in the right y axis and were obtained by the MTT assay cytotoxicity readings after 72 h exposure of drugs to the respective cell line and analyzed by non-linear regression using GraphPad Prism version 8.0 (GraphPad Software, USA) from three independent experiments performed in duplicate.

In order to characterize the anticancer activity of CA_5_ and CA_6_ in TBX2-driven cancers, we first assessed their cytotoxicity in melanoma, breast carcinoma and rhabdomyosarcoma cell lines. These cell lines were selected based on previous data showing that TBX2 plays an important role in their proliferation and survival (Peres et al., 2010; Wansleben et al., 2013; Zhu et al., 2016). Briefly, the cells were treated with a range of concentrations of CA_5_ and CA_6_, for 72 h followed by MTT assay. Regardless of the origin of the cancer, the IC_50_ values obtained for both compounds were in the nanomolar range but the IC_50_ values obtained for CA_5_ were lower (Figure 2 and Table 1). These results suggest that both chromomycins are highly cytotoxic and that CA_5_ was more potent than CA_6_ in this cell panel (Table 1). It is worth noting that, among the cell lines tested, those derived from melanomas were the most sensitive to both chromomycins. Indeed, the IC_50_ values obtained for the melanoma cell lines ranged from 0.3 to 0.8 nM for CA_5_ and from 2.0 to 4.2 nM for CA_6_. Figure 2 shows data where the relative levels of basal TBX2 in all cell lines tested were plotted against IC_50_ values for CA_5_ and CA_6_. The results show that the T47D cells showed slightly higher levels of TBX2 and there is no significant correlation between IC_50_ values and TBX2 levels.

**Table 1:** Cytotoxic activity of chromomycins A_5_ (CA_5_) and A_6_ (CA_6_) against different tumor cells.

**Cell line**	**Compound IC**_****50****_ **(nM) 72 h (95% confidence interval)**	
	**CA_**5**_**	**CA_**6**_**
**MELANOMA**		
501-mel	0.8 (0.6–1.0)	2.0 (1.6–2.4)
WM293A	0.3 (0.2–0.5)	3.2 (2.4–4.3)
MM200	0.5 (0.3–1.0)	4.2 (2.2–8.0)
**BREAST CARCINOMA**		
MCF-7	2.1 (1.8–2.5)	6.5 (5.0–8.4)
T47D	6.5 (5.1–8.9)	6.8 (2.8–16.3)
**RHABDOMYOSARCOMA**		
RD (Embryonal)	3.0 (2.8–3.3)	7.2 (5.6–8.8)
RH30 (Alveolar)	3.5 (3.3–3.6)	5.5 (3.6–7.3)

*The compounds were incubated during 72 h, and cell viability was measured through the MTT assay. IC_50_ and the 95% confidence interval were obtained through non-linear regression using GraphPad Prism version 8.0*.

Other chromomycins were previously reported for their anticancer activities with similar potencies as those described here. Indeed, Toume et al. (2014) reported that CA_2_ (a stereoisomer of CA_5_) and CA_3_ (a stereoisomer of CA_6_) are cytotoxic against a human gastric adenocarcinoma cell line, with IC_50_ values ranging from 1.7 to 22.1 nM. Pettit et al. (2015) also assessed the cytotoxicity of CA_5_ against a mini-panel of human cancer cell lines and found IC_50_ values ranging from 0.6 (MCF-7) to 3.4 nM (KM20L2, colon cancer cell). An earlier investigation from our group also evaluated the cytotoxicity of CA_2_ against a panel of tumor cells from different origins, including colon, prostate, leukemia and melanoma. Consistent with the current study, we showed that the melanoma cells were among the most sensitive to CA_2_. However, CA_2_ had an IC_50_ of 18.8 nM in the metastatic melanoma cell line Malme-3M and was thus less cytotoxic than the CA_5_ and CA_6_ chromomycins tested in this study against melanoma cells (Guimarães et al., 2014). Furthermore, a recent study from our group compared the cytotoxicity of 4 different chromomycins in a 5 human tumor cell line panel and showed that CA_5_ was the most potent, especially against the MM200 (IC_50_ of 0.2 nM) and 501Mel (IC_50_ of 0.8 nM) melanoma cells (Pinto et al., 2019).

**Figure 3:**
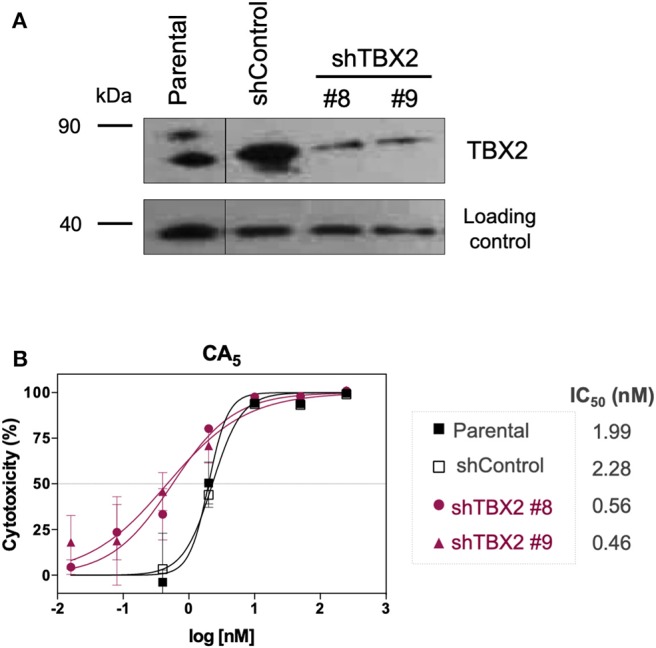
Cytotoxicity of CA_5_ on stably TBX2-knockdown cells (shTBX2). **(A)** TBX2 protein expression in parental, shControl and shTBX2 clones #8 and #9 501mel cell models, achieved by Western blot. **(B)** Cytotoxicity profiles of 501mel cell models exposed to CA_5_. IC_50_ (nM) values for CA_5_ in the 501mel cell models. Cytotoxicity curves and respective IC50 values we obtained by MTT assay, after cells were exposed to the respective drugs during 72 h and calculated by non-linear regression on GraphPad Prism 8.0 (GraphPad Software, USA) from three independent experiments performed in quadruplicate.

To investigate whether CA_5_ exerts its cytotoxicity, in part, through binding TBX2, we firstly tested the effect of knocking down TBX2 on the sensitivity of 501mel cells to the chromomycin. Results from MTT assays show that depleting TBX2 reduced the IC_50_ of CA_5_, by at least 4 times in 501mel cells (Figure 3).

**Figure 4:**
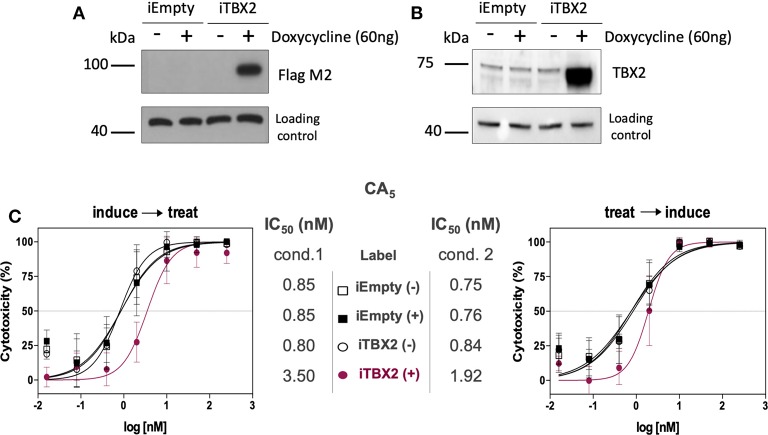
Cell viability assay (MTT) of CA_5_ in 501mel cells with induced and non-induced TBX2-overexpression. **(A,B)** Western blot membranes of samples from iEmpty and iTBX2 cells, without (−) and with (+) 60 ng/mL doxycycline, showing that only iTBX2 (+) is inducing TBX2 overexpression; A—probed for anti-Flag M2; B—probed for anti-TBX2. **(C)** CA_5_ cytotoxicity profiles and respective IC_50_ (nM) values accessed under cond.1 and cond.2 after 48 h drug exposure. IC_50_ values were obtained by non-linear regression using GraphPad Prism version 8.0 (GraphPad Software, USA) from three independent experiments performed in duplicate. Cond.1, experiment setup where induction of TBX2 expression was done before drug treatment; Cond.2, experiment setup where drug treatment was done prior to TBX2 induction.

Furthermore, when TBX2 expression was induced (iTBX2) in 501mel cells genetically engineered to express TBX2 in the presence of doxycycline for 12 h prior to CA_5_ treatment for 48 h, the cells were more resistant to the drug (Figure 4A). When the same experiment was carried out but TBX2 was induced for the last 12 h of CA_5_ treatment, the cells were also more resistant to CA_5_ when compared to their iTBX2-empty counterparts (Figure 4B). Indeed, the IC_50_ values increased by 7 times (from 0.6 to 4.2 nM) when TBX2 was induced prior to CA_5_ treatment and by 1.8 times (from 0.8 to 1.4 nM) when TBX2 was induced post CA_5_ treatment (Figure 4C). The induction of TBX2 prior to exposure to CA_5_ thus produced a greater resistance response in TBX2-expressing cells.

Taking together, the evidences herein suggest that TBX2 is indeed conferring resistance to these drugs, as observed to cisplatin (Davis et al., 2008; Wansleben et al., 2013). Although these findings point to a direct association between TBX2 levels and CA_5_ cytotoxicity, the previously observed relation between TBX2 and DNA repair pathways (Wansleben et al., 2013) should also be taken in account to explain the increased toxicity observed in TBX2-knockdown cells.

In summary, CA_5_ and CA_6_ displayed high levels of cytotoxicity at relatively low concentrations against all TBX2-driven cancer cell lines tested. Taken together, the evidences generated in the present study through reverse affinity chromatography, and MST assays, then pondered with data from the literature, suggest that, beyond the already established DNA-damaging effects, chromomycins, and especially CA_5_, bind TBX2 and its modulation may contribute to the observed cytotoxic properties of this group of molecules.

## Data Availability Statement

The datasets generated for this study are available on request to the corresponding author.

## Author Contributions

All authors listed have made a substantial, direct and intellectual contribution to the work, and approved it for publication.

### Conflict of Interest

The authors declare that the research was conducted in the absence of any commercial or financial relationships that could be construed as a potential conflict of interest.
